# Psychometric validation of the Arabic version of the GAD-7 among Lebanese adolescents

**DOI:** 10.1371/journal.pone.0329627

**Published:** 2025-08-05

**Authors:** Myriam El Khoury-Malhame, Souheil Hallit, Maria-Jose Sanchez-Ruiz, Sleiman El Hajj, Rita Doumit

**Affiliations:** 1 Department of Social and Education Sciences, School of Arts and Sciences, Lebanese American University, Beirut, Lebanon; 2 School of Medicine and Medical Sciences, Holy Spirit University of Kaslik, Jounieh, Lebanon; 3 Psychology Department, College of Humanities, Effat University, Jeddah, Saudi Arabia; 4 Applied Science Research Center, Applied Science Private University, Amman, Jordan; 5 Department of Personality Psychology, Assessment, and Psychological Treatment, School of Psychology, UNED, Madrid, Spain; 6 Department of English and Creative Arts, School of Arts and Sciences, Lebanese American University, Beirut, Lebanon; 7 Alice Ramez Chaghoury School of Nursing, Lebanese American University, Byblos, Lebanon; Alexandria Medicine: Alexandria University Faculty of Medicine, EGYPT

## Abstract

**Introduction:**

Anxiety is one of the major global mental health concerns, particularly amidst accumulating adversities. It is the leading cause of distress in adolescents worldwide and has a profound deleterious impact on their mental and physical health and wellbeing. This paper seeks to identify and validate the psychometric properties of the Arabic version of the GAD-7 in Lebanon, aiming to improve the much-needed overall mental health screening in Middle Eastern countries.

**Methods:**

This study includes a cross-sectional design including 638 adolescents in Lebanese public schools. Participants aged 15−18 years were assessed using GAD-7 (anxiety), PCL-5 (post-traumatic stress disorder), and PTGi (post-traumatic growth) in their Arabic versions at two-time points, spaced three months apart.

**Results:**

Our analyses revealed that the 7 items of the GAD-7 converged into a single factor. Composite reliability of scores was adequate in the total sample (ω = .88/ α = .87). The convergent validity for this model was satisfactory. Results showed invariance across gender at the configural, metric, and scalar levels, with males showing a higher level of wellbeing compared to females. The pre-posttest assessment for the GAD-7 scale was conducted on 359 participants; the intraclass correlation coefficient was adequate 0.83 [95% CI.79;.86]. Our analyses also show that anxiety symptoms were significantly correlated with higher PTSD (*r* = 0.68; *p* < .001) and lower PTG (*r* = −.12; *p* = .004).

**Conclusion:**

The Arabic GAD-7 among Lebanese adolescents displayed highly satisfactory psychometric properties, underscoring its validity. This scale could be valuable for educators and clinicians as a screening tool to rapidly detect anxiety among this vulnerable age group as GAD-7 is easy-to-use, easy to understand, culturally sensitive for Arab population and age appropriate for 15–18-year-old students.

## Introduction

Anxiety disorders represent a prevalent mental health challenge globally, affecting people of all ages and backgrounds. In particular, during adolescence and young adult years, adolescents are prone to mental health problems [1]. Anxiety disorders can significantly impair an individual’s ability to function in daily life, impacting relationships, academic performance, and overall wellbeing [2]. According to the World Health Organization, Generalized Anxiety Disorder (GAD) is a mental health condition characterized by excessive and persistent worry or anxiety about various aspects of life, such as work, relationships, health, or everyday situations [1]. The DSM-5 defines general anxiety disorder as an excessive worry about particular events or performance occurring on most days than not, for more than six months [3]. The worry is difficult to control and is often accompanied by symptoms such as restlessness, irritability, muscle tension, difficulty concentrating, and sleep disturbances [1].

Despite the significant impact of anxiety disorders on the adolescent population, these are often under-diagnosed and untreated [1]. Recently, it has been suggested that care might be improved if clinicians used standardized questionnaires to identify patients with previously unrecognized anxiety disorders. A systematic review of screening tools conducted by Herr et al., [4] revealed that the GAD-7 had the best performance characteristics for identifying anxiety disorders in comparison to other measures. This has also been reported in more recent studies [5] where GAD-7 stands out for its brevity and practical utility. With only 7 items, GAD-7 is easier to use than the Pediatric Anxiety Rating scale (50 items) [6], the State-Trait Anxiety Inventory for Children (20 items) [7] and the Revised Children’s Manifest Anxiety Scale (49 items) [8]. GAD- 7 has strong psychometric properties [9], minimal overlap with scales measuring mental distress and might be completed by an adolescent in less than two minutes [10,11]. In addition, GAD-7 can be employed as a standardized cross-cultural measurement tool as it has been used in the adolescent population in many countries such as Spain [12], Hong Kong [13], China [14] and South Africa [15] among others. The GAD-7 has even been used in the clinical adolescent population with migraine [16] and Type-1 Diabetes [17]. However, despite its widespread use, there is a gap in validation studies among the Arab-speaking adolescent population in particular in Lebanon.

Lebanon is a Middle Eastern low-income country, grappling with political corruption, social unrest, COVID-19 health crisis and more recently the devastating Beirut port explosion in 2020 [18]. The accelerated depreciation of the local currency has further impoverished the middle class, with approximately 50% of households living below poverty lines [19]. Among the most vulnerable at the center of this reality is the youth segment, constituting around 30% of the overall population [20]. Unfortunately, Lebanese adolescents have limited access to mental health support and counseling services [21]. A study conducted among Lebanese children and adolescents in the aftermath of the Beirut blast revealed alarming prevalence rates of probable anxiety among 64% and Post- Traumatic Stress Disorder (PTSD) among 52% of the youth population, which is higher than nonetheless elevated numbers reported in Lebanese adults [22] and other neighboring Arab countries [23]. A recent systematic review to validate screening tools for anxiety disorders and PTSD in Low to Middle Income Countries revealed that the age distribution among screening tools was heavily biased towards the adult population and that children and adolescents accounted for only six of 58 validation studies for anxiety and depression [24]. This systematic review emphasized the urgent need for research focused on identifying anxiety and PTSD disorders among pediatric and adolescent populations, especially in regions marred by civil unrest and conflict.

Therefore, this study seeks to investigate the validity of the Arabic version of GAD-7, a concise and widely used scale in clinical and research settings, as a screening tool for anxiety symptom in adolescents. The primary aim is to analyze the factor structure, reliability and concurrent validity of the Arabic GAD-7 in a sample of Lebanese school students. To better assess its psychometric properties, additional variables related to overall context of trauma accumulation in the country were used, including scales for post-traumatic stress and post-traumatic growth. Trauma symptom severity is indeed known to positively correlate with anxiety [25], whereas post-traumatic growth was documented to negatively correlate with anxiety [26]. As such, GAD-7 would help identify people struggling with anxiety, who would altogether have higher trauma symptoms and reduced capacity for growth.

## Methods

### Procedure

The study received administrative approval from the Lebanese Ministry of Education and Higher Education (MEHE) and was approved by the Institutional Review Board (LAU IRB; reference number: LAU.SAS.MJ1.28/Oct/2021). The recruitment period for this study started on May 1st, 2022 and ended on May 31st, 2023. In accordance with the Declaration of Helsinki, all participants and their parents approved and signed the informed consent. The document was sent home to their parents to sign it while adolescents signed their consent form in class. The consent was given by the Institutional Review Board LAU.SAS.MJ1.28/Oct/2021.

In addition to age and gender, participants reported their parents’ job occupations. They then filled standardized scales for anxiety, PTSD, and PTG used in their validated Arabic versions. The Arabic versions of three scales have been validated in Arabic among Arabic-speaking populations. [27–30]

### Participants

The sample consisted of 701 high school students aged between 14 and 18, who were recruited from four public schools in Lebanon. Schools were assigned randomly by MEHE according to few set criteria for the study in line with funder regulations (public schools, centrally located in the capital or major proximal cities and having boys and girls). Among those participants, 638 students (mean age 16.19 ± 1.02 years) had complete data and were included in the final sample with 56.4% females. A subsample of 359 students (56% of the initial sample) was retested at three months, post-initial assessment, to identify additional psychometric properties of the scale and better investigate the stability of the measurement on longer periods of time.

### Questionnaires

#### GAD-7 (The Generalized Anxiety Disorder).

This is a self-reported questionnaire designed to assess anxiety during the previous two weeks [31]: It is based on seven items “e.g., Feeling nervous, anxious, or on edge” on a four-point Likert scale ranging from 0 “Not at all” to 3 “Nearly every day”. Higher scores indicate higher severity of symptoms, as scores between 0–4 indicated no anxiety, 5–9 mild, 10–14 moderate, and 15 or more severe anxiety symptoms. It has satisfactory internal consistency (α from.79 –.91;) and has been validated among an Arabic Lebanese population [27].

#### PCL-5 (The post traumatic stress disorder checklist for DSM-5).

This is a self-report measure that evaluates PTSD symptoms after a distressing event [32]: Participants answer four Likert scale questions ranging from 0 “Not at all” to 4 “Extremely”; for example, “In the past month, how much have you been bothered by [event]?” High scores indicate higher PTSD symptoms with a conservative cut-off of 6 for severe symptoms. PCL-5 has been shown to have high internal consistency for the English version (α = .95), and has been validated in Arabic-speaking populations [28].

#### PTGI-SF (Posttraumatic growth inventory – short form).

This 10-item scale assesses the experience of positive change resulting from traumatic events [33]: It includes five factors: personal strength, spiritual change, new possibilities in life, appreciation of life, and relating to others. Statements are rated from 0 (“I did not experience this change”) to 5 (“I experienced this change to a great degree”), for example, “I have changed my priorities about what is important in life”. PTGI-SF has shown to have high internal consistency (α = .925) and has been validated in Arabic among Arabic-speaking samples [29,30].

### Analytic strategy

#### Confirmatory factor analysis.

There were no missing responses in the dataset. We used data from the total sample to conduct a CFA using the SPSS AMOS v.29 software. We aimed to enroll a minimum of 140 adolescents following the recommendations of Mundfrom and her colleagues of 3–20 times the number of the scale’s variables [34]. Parameter estimates were obtained using the maximum likelihood method. Multiple fit indices were calculated: root mean square error of approximation (RMSEA), standardized root mean square residual (SRMR), Tucker-Lewis Index (TLI) and Comparative Fit Index (CFI). Values ≤ .08 for RMSEA, ≤ .05 for SRMR, and ≥.90 for CFI and TLI indicate a good fit of the model to the data [35]. Additionally, values of the average variance extracted (AVE) ≥.50 indicated evidence of convergent validity [36]. Multivariate normality was not verified at first; therefore, we performed a non-parametric bootstrapping procedure. To examine the gender invariance of GAD scores, we conducted multi-group CFA using the total sample [37]. Measurement invariance was assessed at the configural, metric, and scalar levels [38]. We accepted ΔCFI ≤ .010 and ΔRMSEA ≤ .015 or ΔSRMR ≤ .010 as evidence of invariance [37].

Composite reliability was assessed using McDonald’s ω and Cronbach’s α, with values greater than.70 reflecting adequate composite reliability [39]. Normality was verified since the skewness and kurtosis values for each item of the scale varied between −1 and +1 [40]. Pearson test was used to correlate the GAD-7 scores with the other scales in the survey. Student t-test was used to compare two means. The intraclass correlation coefficient (ICC) was used to assess the reliability of the scale pre and post, with values higher than 0.75 indicating good reliability [41].

## Results

### Descriptive statistics

The mean and standard deviation of the scores were as follows: GAD-7 anxiety (pre: 9.94 ± 5.43, post: 9.82 ± 5.42), PCL-5 PTSD (62.29 ± 7.80) and PTG (29.38 ± 9.53). In the final sample, 19.6% of adolescents had no anxiety, whereas 33.7%, 24.8% and 21.9% of them showed mild, moderate, and severe anxiety respectively.

#### Confirmatory factor analysis of the GAD scale.

CFA indicated that fit of the one-factor model of the GAD scale was acceptable: RMSEA = .085 (90% CI.067,.104), SRMR = .035, CFI = .966, TLI = .949. The standardized estimates of factor loadings were all adequate S1 Fig. Composite reliability of scores was adequate in the total sample (ω = .88/ α = .87). The convergent validity for this model was satisfactory, as AVE = .51.

#### Gender invariance.

Results showed invariance across genders at the configural, metric, and scalar levels in Table 1. Females showed a higher level of anxiety compared to males (10.96 ± 5.42 vs 7.98 ± 4.94; *t*(636) = −7.25; *p* < .001).

**Table 1:** Measurement invariance of the generalized anxiety disorder 7-item scale across gender in the total sample.

Model	CFI	RMSEA	SRMR	Model Comparison	ΔCFI	ΔRMSEA	ΔSRMR
Configural	.967	.057	.046				
Metric	.968	.051	.050	Configural vs metric	.001	.006	.004
Scalar	.956	.055	.053	Metric vs scalar	.012	.004	.003

*Note.* CFI = Comparative fit index; RMSEA = Steiger-Lind root mean square error of approximation; SRMR = Standardised root mean square residual.

#### Interclass correlation.

The pre-posttest for the GAD-7 scale was done on 359 participants. The intraclass correlation coefficient was adequate = 0.83 [95% CI.79;.86

#### Concurrent validity.

Higher PTSD scores (*a* = .68; p < .001) correlated with higher GAD-7 scores, whereas higher PTG scores (*r = −.12; p = .004*) correlated with lower anxiety.

## Discussion

This study aimed to investigate the psychometric properties of a rapid easy-to-use self-administered anxiety scale in Lebanese adolescents. The GAD-7 emerged from this study as a favorable research-friendly and culturally-adapted option for assessing anxious symptomatology in the targeted age group between 15 and 18 years. We have investigated factor loading onto a single dimension, along with convergent and divergent validity, test-rest reliability as well as measurement invariance across gender at various levels. Taken together, these properties support the use of GAD-7 in the context of Arabic-speaking adolescents, or adolescents facing similarly challenging socio-economic conditions and unstable political situations.

First and foremost, our results indicate alarming levels of anxiety in Lebanese schooled adolescents. In fact, around 80% of participants had detectable symptoms of anxiety, with 47% scoring above moderate to severe cut-offs for probable psychopathology. These excessive rates align with similar findings from a recent study post-Beirut explosion equally reporting elevated levels of anxiety (64%) and PTSD (52%) [42]. These results also echo international concerns about the worsening mental health of adolescents and young adults, especially since the onset of the COVID-19 pandemic [2,43]. Anxiety is the leading cause of emotional dysfunctions and mental illness [44], impacting one-fifth of young individuals [45,46]. In addition to the COVID-19 pandemic and Beirut blast, Lebanon has been facing a crushing economic crisis with the highest inflation rate the country has known in over three decades [47]. Those indescribable events have severely impacted the Lebanese youth. A recent study conducted among Lebanese university students aged between 18 and 35 years showed that significant rates of dissociative experiences and their sub-manifestations (amnesia/depersonalization and absorption) were found among Lebanese university students, with remarkable co-occurrence of a traumatic/stressful pattern, whether on an individual (history of PTSD) or a collective level (Post-traumatic manifestations from Beirut blast, COVID-19 pandemic and/or economic crisis), or whether correlated to an acute single event or to certain chronic stressors, or even to a personal history of depression [48].

This moreover highlights the long-standing mental health burden of piling hardships and calls for public policies and subsequently calls for improved concerted efforts to address the critical needs of underprivileged communities amidst accumulating collective challenges in the country [25,47].

These findings indicate satisfactory psychometric properties of the Arabic version of GAD-7 among Arab-speaking Lebanese adolescents, aligning values and indicators below rigorously acceptable norms. Notably, all 7 items all loaded onto a single factor in our model, similar to the original scale validation [22], and other documented validation studies in both adult [48,49] and adolescent samples [50–52]. The unidimensional structure indicates the presence of a single homogenous underlying factor triggering the multiplicity of symptomatic expression of anxiety, and encompassing aspects of physiological restlessness, cognitive and emotional hyperactivity, and a sense of uncontrollability. The recent surge of validation studies within the past couple of years indicates the rising interest of the scientific community in detecting and assessing the early onsets of anxiety, as symptoms emerging in childhood and adolescence seem to worsen adult prognosis if left untreated [53,54]. Our results point towards the unidimensional structure of the GAD-7 and support its use to assess anxiety in adolescents in the Middle East and contribute to better monitoring the ongoing global mental health crises.

In addition, our study reveals this scale also has measurement invariance across gender at the configural, metric, and scalar levels. While girls in our study scored higher on average than boys on the overall anxiety scale, both girls and boys had equal performances in terms of psychometric properties of the scale items. This is in line with previous literature reviews documenting on one hand the increased levels of anxiety in females as compared to males, [51,55] and on the other, the versatile applicability of GAD-7 scales to both males and females irrespective of the scores and of the manifestation of gender differences of certain symptoms [49,50,56]. Additionally, this study demonstrates robust test-retest reliability over two-time points, with more than 50% of the original student pool tested twice, three months apart. This further supports the psychometric robustness of the GAD-7 in young Arabic-speaking school students.

Lastly, concurrent validity of the 7-item anxiety scale was further matched against other scales assessing mental distress and wellbeing. The GAD-7 had already been used in adolescents screened positive for depression in primary care and demonstrated strong convergent and discriminant validity [57]. In this study, the GAD-7 anxiety scores were correlated to responses post-trauma, given the overall socio-political and health situation in the country. The GAD-7 correlated positively with PTSD and negatively with PTG. These findings have been systematically replicated in studies documenting the parallel increase in anxiety and PTSD symptoms after collective traumatic exposure, [56,58] and similarly in situations such as COVID-19 pandemic, political instability [59], and economic frailty [46,60]. Some studies have pointed to the diminished capacity for growth in people struggling with anxiety, in the absence of additional social support and resilience resources [61,62].

### Clinical implications

This study provides evidence of the efficacy of the GAD-7 scale in assessing anxiety symptoms among Arabic-speaking adolescents in public schools in Lebanon. The user-friendly nature of this scale, as well as its cultural sensitivity and appropriateness for ages 15–18, renders it a valuable tool for educators and clinicians seeking to swiftly assess psychopathological dysfunction. This is particularly important as a screening tool to fine-tune early detection of anxiety in this vulnerable age group. Such early identification is crucial for implementing timely protective interventions among high-school students, empowering them to better navigate upcoming developmental and environmental challenges they face. Consequently, schools can adopt a comprehensive mental health framework that includes periodic GAD-7 screening, robust in-school support, streamlined referral system and treatment of severe cases. Implementing periodic universal screening using the GAD-7 could involve administering the scale at key transition points, such as the beginning of the academic year or before major exam periods, when anxiety levels might be elevated. Counselors, alongside school psychologists and nurses, can be trained to administer the GAD-7, interpret scores, and engage students in follow-up conversations based on their responses. For instances, systematic psychoeducational group workshops centered on stress management could be offered as part of the curriculum to address prevalent levels of anxiety. The GAD-7 regular assessments will also allow school counselors to offer targeted individual support for those reporting severe symptoms and track progress of various interventions.

Our findings also point to the usefulness of using this scale for longitudinal assessment of prevalence rates of anxiety over time. This is particularly important as anxiety in adolescents is associated with impaired functioning, diminished academic performance, heightened dropout rates, as well as lower wellbeing and poorer quality of life [12].

### Limitations and recommendations for future research

This study supports the use of the GAD-7 Arabic version in young adolescents in a Middle Eastern country. However, additional studies are needed to properly generalize these findings to other Arabic-speaking adolescents from diverse socio-educational backgrounds, including those who are not enrolled in schools. Targeting clinical populations with diagnosable mental distress or physical conditions would further enrich our understanding of the scale’s efficacy. Relying solely on self-report measures could introduce biases such as social desirability, memory inaccuracies, or potential misinterpretations of items, and validation studies using only typical-performance tests are at risk of mono-method biases; thus, future studies could benefit from incorporating objective measures of anxiety (such as neurophysiological testing). In addition, it would be beneficial to continue monitoring the applicability of the scale in relation to other demographic factors, such as nuanced Arabic dialects or ways of life in urban v/s rural settings.

Finally, due to the study focus on school adolescents, the findings should be generalizable with caution due to potential selection bias. Future studies should also add a comparative anxiety measure and test for conversion validity.

## Conclusion

The Arabic GAD-7 shows solid psychometric properties in a sample of Lebanese adolescents, further evidencing the valid use of this fast, publicly available, assessment tool to measure levels of anxiety. In this line, current initiatives have shown preliminary efficacy in decreasing psychological distress and fostering wellbeing through an emotional intelligence training program targeting youth in public schools in Lebanon [63]. With potential benefits for education and research settings targeting Arabic-speaking educated youth, GAD-7 could contribute to routine evidence-based developmentally tailored measurements. By integrating the GAD-7 into routine evaluations, stakeholders in both institutional and governmental spheres can gain insights into the emotional and mental wellbeing of school-aged students, thus facilitating informed decision-making and targeted psychological interventions.

## Supporting information

S1 Figure Standardized loading factors of the Generalized Anxiety Disorder 7-item Scale in Arabic. (TIF)
