# Supplementary figures and images for: Psychometric validation of the Arabic version of the GAD-7 among Lebanese adolescents
Source: PLoS One. 2025 Aug 5;20(8):e0329627. doi: 10.1371/journal.pone.0329627 (PMC12324113; doi:10.1371/journal.pone.0329627)

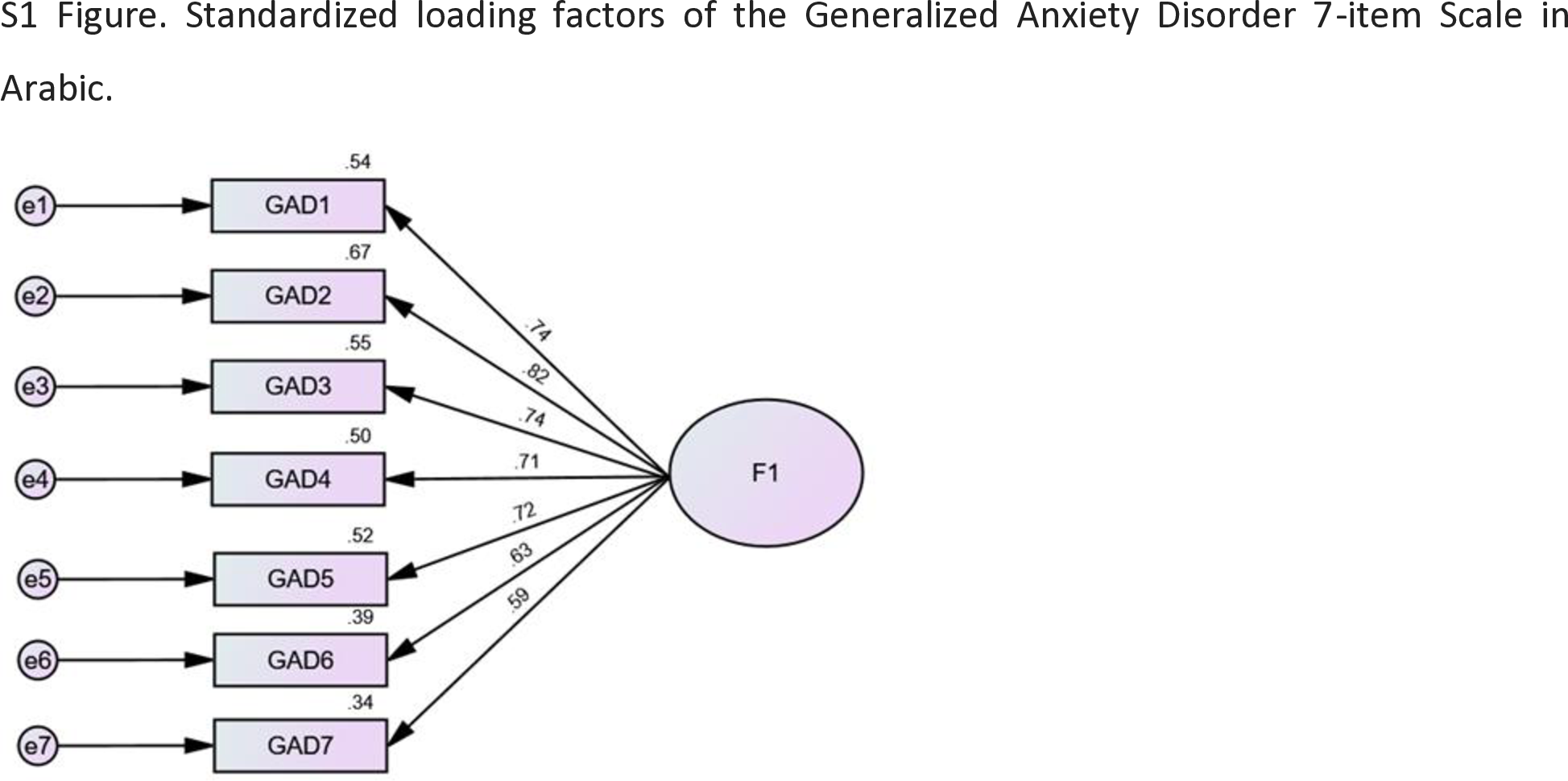

Supplement: S1 Figure — (TIF) [file pone.0329627.s001.tif]
